# Long-term results of anterior cervical corpectomy and fusion with nano-hydroxyapatite/polyamide 66 strut for cervical spondylotic myelopathy

**DOI:** 10.1038/srep26751

**Published:** 2016-05-26

**Authors:** Yuan Zhang, Xu Deng, Dianming Jiang, Xiaoji Luo, Ke Tang, Zenghui Zhao, Weiyang Zhong, Tao Lei, Zhengxue Quan

**Affiliations:** 1 Department of Orthopedic Surgery, The First Affiliated Hospital of Chongqing Medical University, Chongqing, China

## Abstract

To assess the long-term clinical and radiographic outcomes of anterior cervical corpectomy and fusion (ACCF) with a neotype nano-hydroxyapatite/polyamide 66 (n-HA/PA66) strut in the treatment of cervical spondylotic myelopathy (CSM). Fifty patients with CSM who underwent 1- or 2-level ACCF with n-HA/PA66 struts were retrospectively investigated. With a mean follow-up of 79.6 months, the overall mean JOA score, VAS and cervical alignment were improved significantly. At last follow-up, the fusion rate was 98%, and the subsidence rate of the n-HA/PA66 strut was 8%. The “radiolucent gap” at the interface between the n-HA/PA66 strut and the vertebra was further noted to evaluate the osteoconductivity and osseointegration of the strut, and the incidence of it was 62% at the last follow-up. Three patients suffered symptomatic adjacent segment degeneration (ASD). No significant difference was detected in the outcomes between 1- and 2-level corpectomy at follow-ups. In conclusion, the satisfactory outcomes in this study indicated that the n-HA/PA66 strut was an effective graft for cervical reconstruction. Moreover, the osteoconductivity and osseointegration of the strut is still need to be optimized for future clinical application owing to the notably presence of “radiolucent gap” in present study.

First introduced by Smith and Robinson^1^, anterior cervical approaches have remained highly successful for the treatment of cervical spondylotic myelopathy (CSM)^2^. With its advantages of sufficient exposure and thorough decompression, anterior cervical corpectomy and fusion (ACCF) has been advocated for multilevel CSM^3^. After decompression, many struts have been used for the reconstruction of the anterior cervical column. An autogenous bone graft, such as an iliac crest or fibula graft, can achieve a high rate of bony fusion, which is considered the “gold standard” for cervical reconstruction. Unfortunately, donor site complications such as pain, blood loss, hematoma and infection cannot be overlooked^4^,^5^. Allografts have also been used for cervical reconstruction to avoid donor site complications but have been associated with decreased arthrodesis rates, increased graft collapse rates^6^. Titanium mesh filled with autograft from the resected cervical vertebrae for reconstruction has been widely used in recent years with reported fusion rates from 97% to 100%^7^,^8^. However, high rates of subsidence, stress shielding and radio-opacity have been widely reported in previous studies^9^,^10^.

Hydroxyapatite (HA) ceramics have been used as a bone repairing materials because of their biocompatibility and bioactivity. However, the insufficient toughness of HA ceramics restricts their clinical application. To enhance the strength and reduce the brittleness of HA ceramics, the idea of adding HA to polymers was introduced by Bonfield and his collegues in the 1980s^11^. And the hydroxyapatite-reinforced polyethylene (HAPEX) was the first HA/polymer composite that was applied clinically as an implant in the 1990s^12^,^13^. In recent years, various composite materials comprised of inorganic HA and organic polymers have been explored as bone substitutes. Nano-hydroxyapatite/polyamide66 (n-HA/PA66) is a biomimetic composite synthesized from nano-scale HA and the polar polymer PA66 and has been approved for clinical application in recent years. The n-HA/PA66 struts have been used for anterior cervical reconstruction for several years, and satisfactory clinical outcomes have been reported in previous studies^14^,^15^,^16^. However, studies evaluating the long-term results of the n-HA/PA66 strut for ACCF are relatively scarce. In addition, few studies have focus on evaluating the osteoconductivity and osseointegration of the strut itself in clinical applications. This retrospective study involved patients with CSM who underwent anterior cervical corpectomy followed by n-HA/PA66 strut reconstruction with a minimum 5-year follow-up. The purpose of this study was to evaluate the long-term clinical and radiographic outcomes after this procedure.

## Materials and Methods

### Patient selection

This retrospective study was approved by the Institutional Review Board of the First Affiliated Hospital of Chongqing Medical University. The methods used in this study were conducted in accordance with the approved guidelines, and informed consent was obtained from all patients. Between May 2006 and July 2008, a series of 50 consecutive patients (28 men and 22 women) with CSM who underwent ACCF with the n-HA/PA66 strut in our department under the treatment of a single senior surgeon (QUAN) were retrospectively evaluated. All patients exhibited signs and symptoms of myelopathy that corresponded to the radiographic data, including preoperative cervical X-ray films and magnetic resonance images (MRI); these symptoms were refractory to conservative treatment. The exclusion criteria were as follows: patients with cervical trauma, infection, neoplasm, rheumatoid arthritis, severe osteoporosis and previous cervical spine surgery.

### Surgical technique and n-HA/PA66 strut preparation

**Figure 1:**
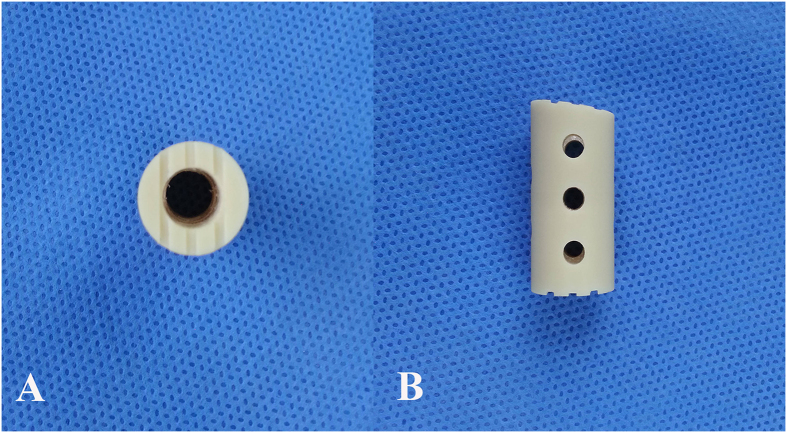
Superior (**A**) and lateral (**B**) views of the nano-hydroxyapatite/polyamide66 strut.

The n-HA/PA66 strut was designed and fabricated by the Institute of Materials Science and Technology, Sichuan University, and our department and was approved for clinical use in 2005 by the State Drug and Food Administration of China. The n-HA/PA66 composite utilized in the present study was synthesized by co-precipitation under normal atmospheric pressure, and the weight ratio of n-HA to PA 66 was approximately 4:6. The composite simulates natural bone in its composition and structure. The organic PA66 macromolecular network uniformly covers the nano-sized HA particles, similar to the interactions that exist between the components of normal bone. In addition, the n-HA in the composite has a similar crystal structure and size to the minerals of natural and can be observed as a thin needle structure with a size of 5–26.7 nm in diameter and 30–84 nm in length^17^. Previous mechanical test results have shown that the n-HA/PA66 composite exhibits a bending strength of 85 MPa, a tensile strength of 71 MPa and a compressive strength of 106 MPa, which are similar values to those of natural cortical bone (80–100, 60–120 and 50–140 MPa, respectively). Moreover, the modulus of the n-HA/PA66 strut was 4.0 GPa, which is also similar to the bone (3.9–11.7 GPa)^18^. The strut was designed with an 8- to 14- mm outer diameter, a 3- to 8-mm inner diameter and an appropriate length for clinical utilization. Each strut has grooves on both ends and several holes on its wall (Fig. 1).

All patients received general anesthesia and a right-sided anterior cervical approach. After adequate exposure of the lesion segment, a 1- or 2-level corpectomy was performed based on the degree of spinal cord compression from the radiological data. We then removed the adjacent cartilage endplates as completely as possible with a cutting burr and curette and implanted the n-HA/PA66 strut filled with the morselized cancellous bone harvested from the resected vertebrae into the decompressed space. A titanium alloy anterior cervical plate was then used for internal fixation. In this study, semi-restricted plates, which included the Slim-Loc (Johnson and Johnson Co., Depuy Spine Ltd., Raynham, MA, USA) and Zephir plates (Medtronic Sofamor Danek Inc., Memphis, TN, USA), were selected in our series. Blood loss and operative time were noted. All of the patients were instructed to wear a cervical collar for six weeks postoperatively.

### Outcomes assessment

**Figure 2:**
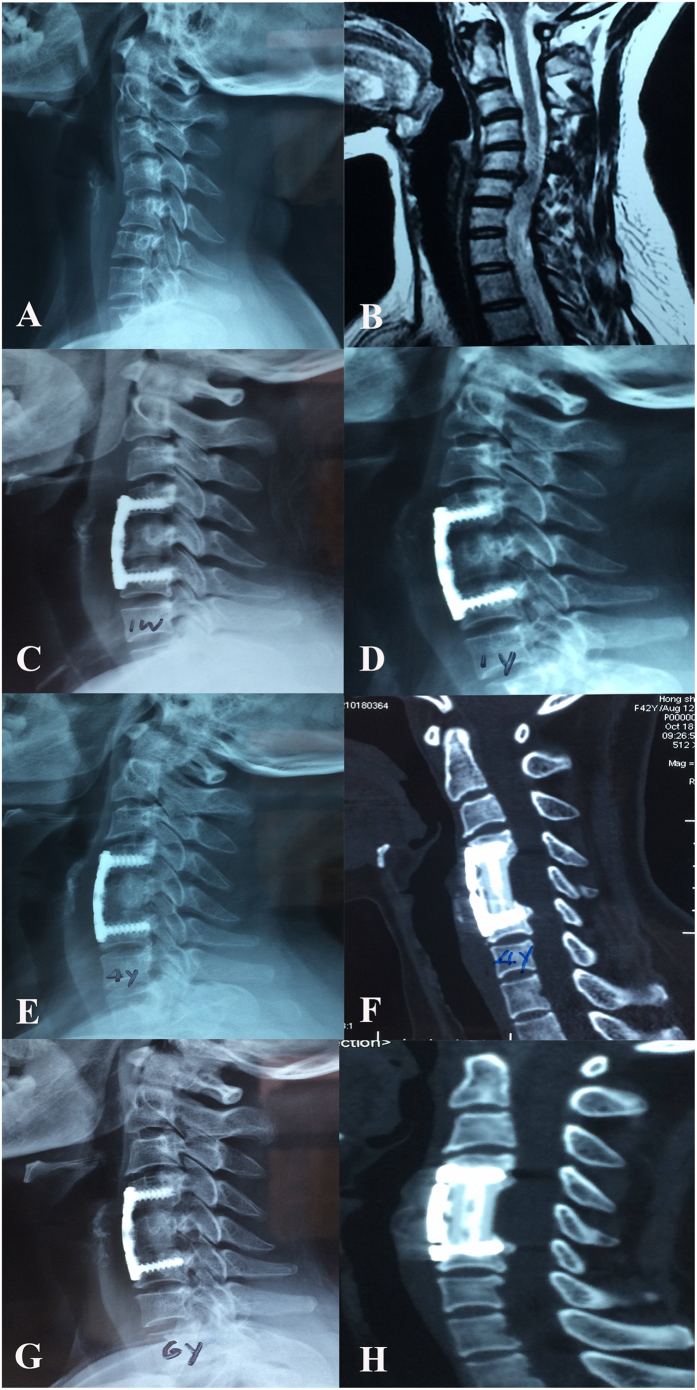
A 38-year-old woman who underwent 1-level corpectomy with a nano-hydroxyapatite/polyamide66 strut for cervical reconstruction. The preoperative cervical X-ray film (**A**) and MRI (**B**) revealed C5/6 disc herniation and segmental cervical kyphosis. The patient underwent a C5 corpectomy and fusion with an n-HA/PA66 strut (**C**). The strut and internal fixation were in position after 1 year of follow-up (**D**). The lateral X-ray film (**E**) and 3D-CT (**F**) scan indicated that the autogenous bone granules filling the strut had achieved bony fusion with adjacent endplates at the 4-year follow-up. The radiographic films revealed satisfying bony fusion with no obvious strut migration or subsidence at the 6-year follow-up ; no radiolucent gap occurred at the interface between the strut and the adjacent vertebrae (**G,H**).

**Figure 3:**
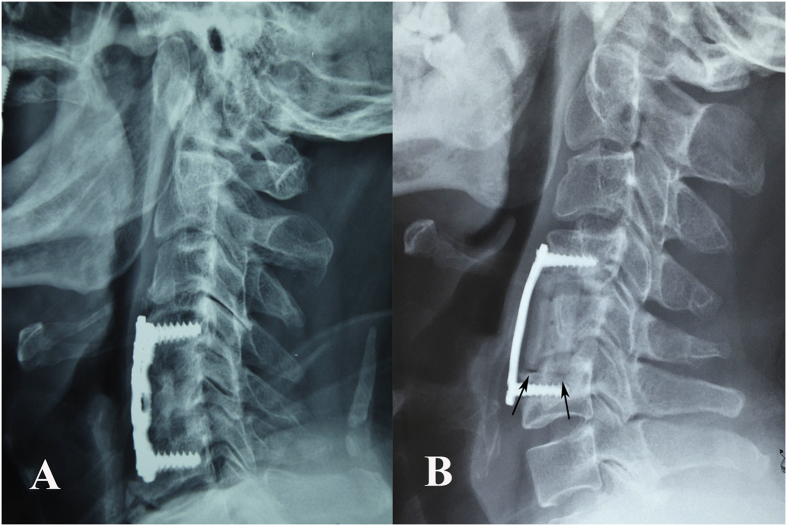
Evaluation of the radiolucent gap between the n-HA/PA66 strut and its contacted endplate. (**A**) No radiolucent gap was observed. (**B**) A radiolucent gap was observed at the conjunction site (black arrows).

Clinical and radiological parameters were collected preoperatively, immediately postoperatively, at the one-year follow-up and then discontinuously until the last follow-up (at least 5 years of follow-up). The Japanese Orthopedic Association (JOA) score was used to assess neurologic status, and the visual analogue scale (VAS) was used to grade arm and neck pain. Lateral cervical radiographs in neutral, flexion and extension positions were collected for all patients before surgery and sequentially at follow-ups to evaluate the radiographic outcomes, including cervical lordosis, fused segmental height, fusion status and the incidence of subsidence. On cervical lateral plain films in the neutral position, the fused segmental height was defined as the distance between the midpoints of the superior endplate of the cephalic vertebra and the inferior endplate of the caudal vertebra in the fused segments. Height loss of the fused segment was measured as the difference between the immediate post-surgery measurements and the follow-up measurements; subsidence was defined as a height loss of more than 3 mm. Cervical lordosis was defined as the angle formed between the lower endplates of C2 and C7 as measured by the Cobb method. Bony fusion was defined as the formation of trabeculation between the bone autograft inside the strut and the adjacent endplates and the absence of motion between the spinous processes within the involved segments on flexion/extension plain radiographs. Three-dimensional computed tomography (3D-CT) scans were also obtained to further confirm fusion status (Fig. 2). We also evaluated and recorded the presence of a “radiolucent gap” between the n-HA/PA66 strut and its contacted endplate on the follow-up plain radiographs to assess the osteoconductivity and osseointegration of the strut (Fig. 3). Symptomatic adjacent segment degeneration (ASD) was defined on the basis of clinical symptoms and homologous MRI examination. Postoperative complications, including hoarseness, dysphagia, C5 nerve root palsy, cerebrospinal fluid leakage, infection, pseudarthrosis, strut migration and internal fixation-related complications, were also recorded.

### Statistical analysis

SPSS 16.0 software (SPSS, Inc., Chicago, IL) was used for statistical analysis. The quantitative data are presented as the mean ± standard deviation. Repeated-measures ANOVA was used for comparisons between pre- and postoperative variables. Independent t-tests were used to compare quantitative data between the two groups. Pearson’s Chi squared test was used for categorical data. Statistical significance was accepted at P < 0.05.

## Results

This long-term retrospective study included 50 patients (28 men and 22 women) with a mean age of 57.5 years (range from 36 to 80 years). The mean follow-up was 79.6 months (range from 64 to 91 months). The average length of hospital stay was 15.6 days, the mean surgery time was 153.8 minutes, and the mean blood loss per patient was 128.2 ml. A 1-level corpectomy and fusion was performed in 39 patients, and a 2-level corpectomy was performed in 11 patients (Table 1).

Clinical outcomes were evaluated by the JOA and VAS scores. The overall mean JOA score was 12.48 ± 1.81 preoperatively and significantly improved to 15.20 ± 1.23 at the last follow-up (P < 0.001). The overall mean VAS was 4.66 ± 1.60 preoperatively and significantly decreased to 1.30 ± 1.13 at the last follow-up (P < 0.001). The evaluation of radiographic outcomes included fused segmental height, C2–C7 Cobb angle, fusion rate, subsidence rate and the incidence of a “radiolucent gap”. The mean fused segmental height was 56.24 ± 9.24 mm preoperatively and significantly improved to 64.12 ± 9.33 mm postoperatively (P < 0.001); this was maintained at 62.39 ± 9.23 mm at the last follow-up. The mean C2–C7 Cobb angle was 9.50 ± 6.07° preoperatively, improved to 13.04 ± 5.22° immediately postoperatively (P < 0.001), and was 11.06 ± 5.28° at the last follow-up. A bony fusion was observed in 46 of 50 cases (92%) at the one-year follow-up and reached 49 of 50 cases (98%) by the last follow-up. Subsidence was defined as a loss of segmental height of more than 3 mm at follow-up compared with the immediate postoperative height. The subsidence rate was 4% (2/50) at the one-year follow-up and increased to 8% (4/50) at the final follow-up. To assess the osteoconductivity and osseointegration of the n-HA/PA66 strut, we recorded the rate of occurrence of a “radiolucent gap” at the interface between the n-HA/PA66 strut and the endplate at the one-year and last follow-up, which were 56% (28/50) and 62% (31/50), respectively. We did not observe a “radiolucent gap” on early follow-up radiographs in three patients, but we did observe a gap at the last follow-up. Three patients (6%) were diagnosed with symptomatic ASD by clinical symptoms combined with MRI at the last follow-up. All three patients received conservative treatment, and the symptoms were controlled effectively. With no progression of neurologic symptoms, none of the patients required further surgical intervention (Table 2).

We further compared the clinical and radiographic outcomes between 1- and 2-level corpectomy. The loss of fused segmental height primarily occurred in the first year after surgery: 1.26 ± 0.64 mm in the 1-level corpectomy versus 1.54 ± 0.61 mm in the 2-level corpectomy (P = 0.204); we observed little progression at the last follow-up, with values of 1.68 ± 0.76 mm versus 1.88 ± 0.60 mm (P = 0.419), respectively. Meanwhile, no significant differences were detected regarding increase in JOA scrore, decrease in VAS score, and the fusion rate, subsidence rate, incidence of symptomatic ASD, or incidence of “radiolucent gap” between 1- and 2-level corpectomy at each follow-up (Table 3).

**Figure 4:**
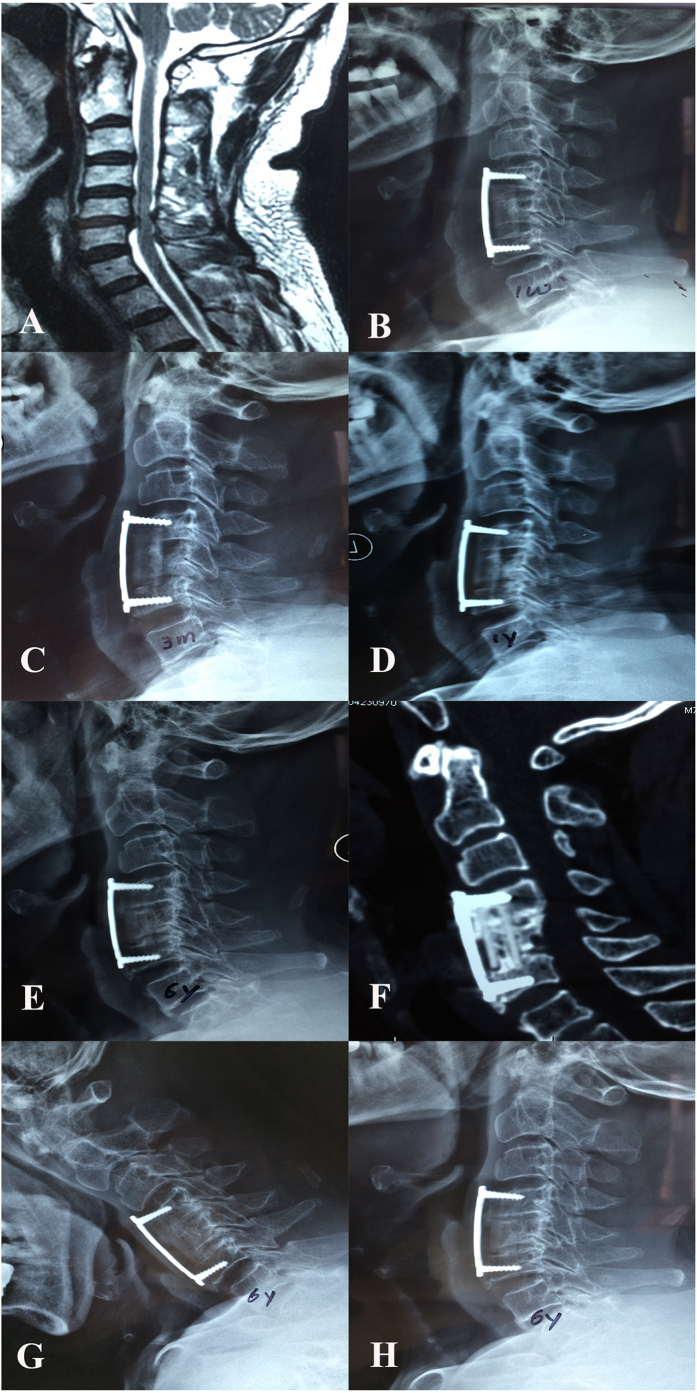
A 73-year old man who underwent 1-level ACCF with an n-HA/PA66 strut. Preoperative MRI (**A**) scans revealed the lesion segment. A cervical lateral X-ray at one week after surgery (**B**) revealed that the strut was in the appropriate position, but the anterior titanium plate was too long. The n-HA/PA66 strut sank into the upper endplate, and internal fixation was dislodged at 3 months after surgery (**C**). At the 1-year follow-up, the subsidence of the n-HA/PA66 strut and the internal fixation migration were aggravated (**D**). A postoperative lateral X-ray (**E**) and 3D-CT (**F**) at the 6-year follow-up revealed bony fusion between the autograft inside the strut and the adjacent vertebrae. Osteophytes were also observed at the gap between the anterior plate and the upper/lower vertebral bodies. The flexion (**G**) and extension (**H**) radiographs revealed solid fusion in the fusion segment.

The postoperative complications observed in our series included C5 nerve palsy in one patient, who was treated with non-steroidal anti-inflammatory drugs (NSAIDS); the symptoms spontaneously resolved six months after surgery. Cerebrospinal fluid leakage was observed in three patients and was successfully treated by drainage and dressing changes. Superficial surgical site infections were observed in two patients and were successfully treated with intravenous antibiotics. Dysphagia was observed in five patients, and the symptoms spontaneously disappeared within two weeks. One patient experienced screw breakage in the caudal vertebra after a 2-level corpectomy; however, bony fusion was observed, and the anterior plate system and n-HA/PA66 strut maintained their positions. Furthermore, no clear signs or symptoms of radiculopathy or myelopathy were observed; thus revision surgery was not performed in this patient. One patient who underwent 1-level corpectomy began to exhibit n-HA/PA66 strut subsidence at the upper endplate 3 months after surgery that was consistent with internal fixation system migration. We attributed these results to intra-operative overdistraction. The patient required no further interventions because of the absence of symptoms. At the follow-ups, we did not observe further deterioration of migration relative to that observed at the 1-year follow-up. Furthermore, bony fusion was observed between the graft and the endplates, and osteophytes were also observed in the fused segment at the last follow-up (6 years after surgery) (Fig. 4).

## Discussion

Anterior cervical corpectomy and fusion (ACCF) is a reliable treatment for cervical spondylotic myelopathy (CSM). The advantages of this procedure are the direct decompression and removal of the elements causing the compression on the cervical spinal cord, including soft disc herniation, osteophytes and ossification of the posterior longitudinal ligament (OPLL)^19^. To ensure relief of the clinical symptoms caused by spinal cord compression, adequate decompression is essential. Furthermore, to achieve long-term stabilization, reconstruction of the cervical spinal column after corpectomy is equally crucial. Regardless of the reconstruction technique used after decompression, solid bony fusion is the ultimate purpose. Autografts have been considered the “gold-standard” for cervical reconstruction after decompression; however, the incidental donor-site complications cannot be overlooked. For these reasons, various graft materials and fusion devices have been developed for cervical reconstruction and have achieved satisfactory results^20^,^21^,^22^,^23^,^24^. In natural bone, the hierarchical organization of calcium phosphate crystals and collagen fibers allows the bone to possess excellent mechanical properties. The n-HA/PA66 strut is a neotype bionic non-metallic implant synthesized from nano-hydroxyapatite and polyamide66 in specific proportions that mimic the constituent form of natural bone and formed by thermal pressing and injection molding technique^17^. The strength and toughness of n-HA/PA66 materials depends primarily on the uniform distribution of stiff nano-HA granules in the soft PA66 matrix. Furthermore, to construct an n-HA/polymer composite with good mechanical properties, the interface between the inorganic n-HA and the organic polymer should be optimized to create proper chemical bonds between the two phases^25^. Previous infrared spectra of the n-HA/PA66 composite have demonstrated that n-HA is linked to PA66 via hydrogen bonds and/or by formation of a carboxyl-calcium-carboxyl linkage ([-COO]-Ca^2+^-[-COO]). These kinds of chemical bonds establish a strong interaction between n-HA and PA66 and result in the good mechanical properties of the n-HA/PA66 composite^18^,^26^. The bending strength, tensile strength, compressive strength and elastic modulus of the n-HA/PA66 strut were 85 MPa, 71 MPa, 106 MPa and 4.0 GPa, respectively, all of which were similar to natural bone^27^,^28^. As described by Wolff’s law, bone grows in response to applied stress and is resorbed if a mechanical stimulus is lacking. It has been reported that the elastic modulus of the titanium mesh is 110 GPa, which is significantly higher than the corresponding value of approximately 12 GPa observed for a bone graft inside the strut. However, the elastic modulus of the n-HA/PA66 cage was founded to be 4.0 GPa, which is substantially lower than that of a bone graft. Theoretically, the n-HA/PA66 strut can avoid the stress shield caused by some metallic implants and promote bony fusion^15^,^16^. Previous studies reported satisfactory clinical and radiographic outcomes of ACCF with the n-HA/PA66 strut in short- and mid-term follow-up. A 94.3% bony fusion rate and a 2.9% subsidence rate were reported in 35 patients in our previous research when using n-HA/PA66 strut for ACCF with an average of 38 months of follow-up^14^. Yang *et al*.^15^ also reported a 97% fusion rate and a 6% subsidence rate in 35 patients with single-level cervical corpectomy followed by n-HA/PA66 strut reconstruction over a mean of 48 months follow-up. However, studies assessing the long-term results of an n-HA/PA66 strut in ACCF are scarce. In this long-term efficacy assessment, satisfactory outcomes, including a 98% fusion rate and 8% subsidence rate at the last follow-up and an early fusion rate of 92% at the one-year follow-up, were observed. These results are consistent with those of previous short-term studies. Moreover, the n-HA/PA66 strut is a nonmetallic implant that is easily penetrated by X-rays. This property permits the ease of evaluation of the fusion status on plain radiographs and CT^29^.

Currently, many of the struts that are used in cervical reconstruction, such as PEEK, and PMMA, are considered to have a nonbiological “bulk” due to the lack of osseointegration^30^. In contrast to these struts, the n-HA/PA66 strut has been validated as a bioactive material with the ability to promote new bone formation and provide a scaffold for osteogenesis^31^. In previous animal experiments, the n-HA/PA66 struts were implanted into the cervical spine of two-year-old goats to evaluate the osteoconductivity and osseointegration of the strut. Radiological and histological osseointegration were observed between the strut and the adjacent cervical vertebra after 24 weeks. These results suggest that n-HA/PA66 is a bioactive material with excellent osteoconductivity and osseointegration^32^. Theoretically, because of the excellent bioactivity of the strut, osseointegration should occur at the interface between the strut and the adjacent endplate. However, in this long-term follow-up study, we specifically assessed the fusion status between the strut and the adjacent endplate by noting the “radiolucent gap” between the strut and the adjacent endplate in the radiographic images to evaluate the osseointegration of the n-HA/PA66 strut itself. Our results indicated that a “radiolucent gap” between the strut and the adjacent endplate was observed in 28 patients (56%, 28/50) at one-year follow-up and in 31 patients (62%, 31/50) by the last follow-up. Interestingly, we also observed that in three cases, bridging of the bony trabeculae had already formed by the short-term follow-up; however, bridging of the bony trabeculae had disappeared, and “radiolucent gap” had appeared by the last follow-up. The main reason for the emergence of the “radiolucent gap”could be the insufficient osteoconductivity of the n-HA/PA66 composite itself. In addition, we also suggest that there are other reasons for the presence of the “radiolucent gap”. First, when we implanted the strut into the cavity between two vertebrae after corpectomy during surgery, the interface between the annular bottom of the n-HA/PA66 strut and the adjacent endplate may not have been completely matched, resulting in angulation at the contact surface and further inducing fibrous tissue to grow into the gap between the strut and endplate. Under these conditions, osseointegration between the strut and the vertebra may have been inhibited; the radiographic penetrability of the fibrous tissue exhibited a “radiolucent gap” after surgery. In addition, in the early stages after surgery, the n-HA/PA66 strut acted as a scaffold at the corpectomy segment and was subjected to the major stresses, which consequently were transformed into mechanical stimuli to promote temporary osseointegration between the annular bottom of the n-HA/PA66 strut and the adjacent endplate. However, along with the bony fusion between the autograft inside the strut and the adjacent vertebra, the mechanical load was transfer from the strut to the autograft, and as a result, the bridging of bony trabeculae between the annular bottom of the n-HA/PA66 strut and the adjacent endplate gradually resorbed due to the lack of mechanical stimuli. Based on our long-term results, we suggest that to ensure the successful achievement of osseointegration between the strut and the adjacent endplate in cervical reconstruction should be depend not only on the characteristics of the strut itself but also on the feasibility of the microenvironment around the strut.

Titanium mesh packed with cancellous bone harvested from the resected cervical vertebrae has been widely used for reconstruction, with numerous satisfactory clinical outcomes reported^8^,^33^. However, titanium mesh subsidence is frequently observed at follow-up, possibly leading to a loss of reconstructive height and cervical lordosis, foraminal stenosis, and the recompression of the spinal cord and nerve roots^34^. Chen *et al*.^35^ reported that subsidence of titanium mesh occurred in 19% of the cases in their study and demonstrated that the subsidence was correlated with poor clinical outcomes. The reasons for subsidence vary and include intraoperative bony endplate preservation, osteoporosis and strut material and shape^36^. However, the sharp footprint of the titanium mesh that led to the small contact surface between the strut and endplate was thought to be the main cause of subsidence^15^,^37^. A previous study reported that broadening the contact surface between the titanium mesh and the adjacent endplate could effectively decrease the incidence of subsidence^38^. In the present study, the n-HA/PA strut was designed with a wide annular rim (nearly three millimeters) to eliminate the risk of subsidence. Moreover, the elastic modulus of the n-HA/PA strut is similar to the natural bone, which could also be beneficial in avoiding subsidence. In this study, the height of the fused segment significantly increased from 56.24 ± 9.24 mm preoperatively to 64.12 ± 9.33 mm postoperatively and was effectively maintained at 62.6 ± 9.4 mm at the last follow-up; the subsidence rate was considerable at 8% (4/50).

Daubs^39^ reported a high early failure rate (75%) for cervical reconstruction after multilevel corpectomies. Two-level corpectomy and graft fusion has been a common longstanding approach for three-level CSM. However, to avoid long segmental corpectomy-related complications, multilevel ACDF (anterior cervical discectomy and fusion) and hybrid constructs (one corpectomy plus one ACDF) have been used for three-level CSM in recent years^40^. Nevertheless, controversy persists concerning which is the best reconstruction approach. Chen *et al*.^35^ illustrated that 2-level corpectomy was more susceptible to severe subsidence compared with 1-level corpectomy and concluded that the number of corpectomy levels was a risk factor for severe subsidence, which might lead to poor clinical outcomes and subsidence-related complications. On the other hand, some studies reported that multi-level ACDF may lead to incomplete limited exposure, decompression and pseudarthrosis as a result of the increased number of bony surfaces^41^,^42^,^43^. In our series using n-HA/PA66 struts for reconstruction, a 2-level corpectomy was not more susceptible to the loss of the fused segmental height than a 1-level corpectomy. Furthermore, we did not detect a lower fusion rate or higher subsidence rate in 2-level corpectomy relative to 1-level corpectomy. All of these data suggest that the n-HA/PA66 strut for ACCF can ensure good biomechanical stabilization even for reconstruction after a 2-level corpectomy. We observed only one patient with screw breakage at the caudal vertebrae after a 2-level corpectomy in our study. However, bony fusion was achieved in the operative segment at the last follow-up, and the patient remained asymptomatic. These results indicate that the n-HA/PA66 strut can provide satisfactory maintenance of fused segmental height that can help decrease the loading on the anterior plate and lower the rate of hardware-related complications. Only one patient in our study suffered strut subsidence at the upper endplate consistent with internal fixation system migration. However, this patient was asymptomatic. Furthermore, no aggravation of the plate migration was noted at follow-up, whereas a bony fusion was observed at the last follow-up. According to our analysis of radiographic data, we concluded that the primary causes of subsidence were intraoperative over-distraction of the adjacent vertebrae and the misuse of an excessively long plate for internal fixation, leading to early instability at the contact face between the strut and the upper vertebra.

Previous studies reported that the incidence of adjacent segment degeneration after anterior cervical fusion ranged from 7% to 15%^44^,^45^. Whether adjacent segment degeneration is the result of the cervical fusion or the progression of natural degeneration remains a matter of controversy^46^. Previous literature suggested that ASD is caused by the dual action of natural and accelerated degeneration of adjacent segments^47^. In the present study, the incidence of symptomatic ASD was relatively low at 6% (3/50). Theoretically, with increased length of the fusion segment, the motion of adjacent segments will increase to compensate, which would increase susceptibility to ASD. However, patients with 2-level ACCF did not exhibit a higher incidence of ASD than patients with 1-level ACCF in present study (9.09% vs. 5.13%, p = 0.534). Yin *et al*.^48^ conducted a meta-analysis comparing cervical disc arthroplasty and anterior cervical discectomy and fusion and concluded that although the cervical disc arthroplasty could retain segmental motion at the operative level, adjacent level motion did not differ after arthroplasty and fusion. These results, combined with our long-term results, lead us to speculate that the progression of the natural degeneration could be the principal cause of ASD. Although the precise mechanism of ASD remains unclear, it is widely recognized that pre-existing adjacent segment degeneration is the main determinant of ASD^49^,^50^. In the present study, all three patients who suffered symptomatic ASD exhibited varying degrees of preoperative degeneration at adjacent segments.

In this long-term study, we observed satisfactory improvement in the JOA score from a mean 12.48 ± 1.81 preoperatively to 15.20 ± 1.23 at the last follow-up. The VAS for neck and arm pain exhibited a significant decline from 4.66 ± 1.60 preoperatively to 1.30 ± 1.13 at the last follow-up. This was similar to Ying’s results with a series of 178 myelopathic patients who underwent anterior decompression and reported an improvement of nearly 2.5 points in the JOA score^51^. In our series, none of the patients had a severe preoperative JOA score; this fact was an important reason for the impressive clinical results. It has been widely accepted that a poor preoperative neurological status is highly predictive of poorer postoperative recovery^52^,^53^,^54^. Thus, early decompression should be performed as soon as spinal cord compression is confirmed by a combination of clinical symptoms and radiographic data. In the present study, the C2–C7 Cobb angle was 9.50 ± 6.07° preoperatively and improved to 11.06 ± 5.28° at the last follow-up. Andaluz *et al*.^55^ demonstrated a significant relationship between postoperative C2–C7 regional kyphosis and chronic neck pain. Therefore, the satisfactory clinical outcomes in this study may also be attributable to the effective improvement and maintenance of cervical alignment.

## Conclusion

This report describes a long-term retrospective study of the utilization of n-HA/PA66 struts for cervical reconstruction after corpectomy. Due to the favorable biomechanical characteristics of the strut, satisfactory clinical outcomes, high bony fusion rates, and acceptable subsidence were achieved. However, the osteoconductivity and osseointegration characteristics of the strut were still insufficient in clinical applications. Furthermore, some limitations to this study. This was a retrospective study with a small sample size, including only fifty patients in the evaluation. In the future, more material research on n-HA/PA66 composite is urgently needed to optimize its osteoconductivity and osseointegration. Furthermore, a prospective, randomized and long-term follow-up study is needed to compare the n-HA/PA66 strut with other graft materials.

## Additional Information

**How to cite this article**: Zhang, Y. *et al*. Long-term results of anterior cervical corpectomy and fusion with nano-hydroxyapatite/polyamide 66 strut for cervical spondylotic myelopathy. *Sci. Rep.* **6**, 26751; doi: 10.1038/srep26751 (2016).

## Figures and Tables

**Table 1:** Demographic data of the patients.

Gender (Male/female)	(28/22)
Age (years)	57.52 ± 10.82
Hospital stay (days)	15.60 ± 3.26
Surgery time (min)	153.80 ± 30.47
Blood loss (ml)	128.20 ± 56.67
Follow-up (mouths)	79.60 ± 6.22
Involved segments	
1-level corpectomy	
C4	7
C5	27
C6	5
2-level corpectomy	
C4-C5	7
C5-C6	4

**Table 2:** Clinical and radiographic outcomes of the study patients.

	Pre-operation	Post-operation	One year follow-up	Last follow-up
JOA score	12.48 ± 1.81	14.78 ± 1.23	15.16 ± 1.09	15.20 ± 1.23
VAS score	4.66 ± 1.60	2.24 ±1.20	1.26 ± 0.96	1.30 ± 1.13
Segmental height (mm)	56.24 ± 9.24	64.12 ± 9.33	62.79 ± 9.20	62.39 ± 9.23
Cervical alignment (°)	9.50 ± 6.07	13.04 ± 5.22	11.70 ± 5.19	11.06 ± 5.28
Fusion rate			92% (46/50)	98% (49/50)
Subsidence rate			4% (2/50)	8% (4/50)
Radiolucent gap			56% (28/50)	62% (31/50)
Symptomatic ASD			0% (0/50)	6% (3/50)

**Table 3:** Comparison of outcomes between 1- and 2-level corpectomy.

	one year follow-up	Last follow-up
Reduction of segmental height (mm)		
1-level corpectomy	1.26 ± 0.64	1.68 ± 0.76
2-level corpectomy	1.54 ± 0.61	1.88 ± 0.60
p value	0.204	0.419
Fusion rate		
1-level corpectomy	92.31% (36/39)	97.44% (38/39)
2-level corpectomy	90.9% (10/11)	100% (11/11)
p value	0.643	0.78
Subsidence rate		
1-level corpectomy	2.56% (1/39)	7.69% (3/39)
2-level corpectomy	9.1% (1/11)	9.1% (1/11)
p value	0.395	0.643
Incidence of symptomatic ASD		
1-level corpectomy	0% (0/39)	5.13% (2/39)
2-level corpectomy	0% (0/11)	9.09% (1/11)
p value		0.534
Incidence of radiolucent gap		
1-level corpectomy	56.41% (22/39)	58.97% (23/39)
2-level corpectomy	54.55% (6/11)	72.73% (8/11)
p value	0.912	0.632
JOA increase		
1-level corpectomy	2.59 ± 1.55	2.67 ± 1.74
2-level corpectomy	3.00 ± 1.00	2.91 ± 1.22
p value	0.413	0.668
VAS decrease		
1-level corpectomy	3.26 ± 1.31	3.21 ± 1.49
2-level corpectomy	3.91 ± 0.83	3.91 ± 1.04
p value	0.126	0.150
